# Serum copeptin in women with gestational diabetes mellitus: A meta-analysis

**DOI:** 10.17305/bb.2024.10114

**Published:** 2024-08-01

**Authors:** Yuanqi He, Xue Li, Xiaoxiao Li, Weiwei Cui, Shihong Zhang

**Affiliations:** 1 Department of Obstetrics, Weihai Municipal Hospital, Cheeloo College of Medicine, Shandong University, Weihai, China; 2 Department of Gynaecology and Obstetrics, Weihai Municipal Hospital, Cheeloo College of Medicine, Shandong University, Weihai, China

**Keywords:** Copeptin, gestational diabetes mellitus (GDM), biomarker, serum, meta-analysis

## Abstract

Previous studies have reported mixed results regarding the relationship between serum copeptin levels and gestational diabetes mellitus (GDM) risk. To address inconsistencies in prior research, this meta-analysis examines the potential link between serum copeptin levels and the risk of developing GDM. Our objective was to comprehensively evaluate this association. We systematically reviewed observational studies from Medline, Web of Science, Embase, Wanfang, and China National Knowledge Infrastructure (CNKI) databases up to October 15, 2023, employing a random-effects model to integrate the data while considering heterogeneity. This analysis incorporated 10 studies comprising 625 women with GDM and 1212 healthy pregnant controls. Our findings showed no significant difference in serum copeptin levels between women with GDM and those without (standardized mean difference [SMD] 0.01, 95% confidence interval [CI] −0.22 to 0.24, *P* ═ 0.92, *I*^2^ ═ 75%). Univariate meta-analysis indicated a positive correlation between the body mass index (BMI) of the participants and the outcomes (coefficient ═ 0.11, *P* ═ 0.002). Further subgroup analysis demonstrated that women with a mean BMI ≥ 26 kg/m^2^ and GDM had significantly higher serum copeptin levels compared to their non-GDM counterparts (SMD 0.31, 95% CI 0.05 to 0.57, *P* ═ 0.02, *I*^2^ ═ 46%). Conversely, no difference was observed in women with a BMI < 26 kg/m^2^ (SMD −0.23, 95% CI −0.37 to −0.09, *P* ═ 0.002, *I*^2^ ═ 0%, *P* for subgroup difference ═ 0.003). Variables, such as the country of study, maternal age, the timing of blood sampling, copeptin measurement methods, or GDM diagnostic criteria did not significantly affect the results. In summary, the association between serum copeptin levels and GDM risk is influenced by the BMI of pregnant women, indicating that elevated serum copeptin might be linked to GDM in individuals with a BMI ≥ 26 kg/m^2^.

## Introduction

Gestational diabetes mellitus (GDM) affects approximately 1%–14% of pregnancies globally [1–4]. Women with GDM have been shown to have higher risks of preterm birth, preeclampsia, instrumental delivery, type 2 diabetes mellitus, and cardiovascular diseases in the future [5–7]. Additionally, infants born to women with GDM may suffer from macrosomia, shoulder dystocia, prolonged labor, postpartum hypoglycemia, and metabolic disorders, such as obesity, impaired glucose tolerance, and early-onset diabetes [5–7]. Consequently, it is imperative to assess the underlying mechanisms involved in the development of GDM.

Copeptin, the C-terminal fragment of arginine provasopressin (AVP), serves as a biomarker for non-specific stress response [8, 9]. It has been acknowledged as a risk factor and prognostic biomarker for cardiovascular diseases [10, 11]. Additionally, serum copeptin has been associated with the development and advancement of type 1 and type 2 diabetes [12]. A preliminary cohort study conducted in Sweden demonstrated that elevated serum copeptin level independently predicts a higher risk for diabetes mellitus in a population derived from the community, regardless of established clinical risk factors [13]. Subsequent investigations have indicated a potential link between the elevated levels of serum copeptin and the incidence of diabetic complications, including diabetic nephropathy and retinopathy [14, 15]. Nevertheless, research examining the relationship between serum copeptin and GDM has yielded conflicting findings [16–25]. Some studies suggested a higher serum copeptin in women with GDM as compared to those without GDM [22, 25], while others did not show the same results [16–21, 23, 24]. Therefore, it remains unknown regarding the association between serum copeptin and GDM. In addition, the mechanisms underlying the inconsistent results of the previous studies are also to be determined. Consequently, the objective of this meta-analysis was to comprehensively assess the association between serum copeptin levels and the likelihood of developing GDM. Particularly, we performed comprehensive meta-regression and subgroup analyses to explore the potential clinical factors that may modify the association between the serum copeptin level and GDM.

## Materials and methods

The Preferred Reporting Items for Systematic Reviews and Meta-Analyses (PRISMA 2020) [26, 27] and the Cochrane Handbook for Systematic Reviews and Meta-analyses [28] were followed in this meta-analysis during study design, data collection, statistical analysis, and result interpretation.

### Literature search

To identify studies relevant to the aim of the meta-analysis, we searched Medline, Web of Science, Embase, Wanfang, and CNKI (China National Knowledge Infrastructure) databases utilizing comprehensive search terms involving: (1) “copeptin” OR “C-terminal provasopressin” and (2) “GDM” OR [(“gestational” OR “pregnancy” OR “pregnant”) AND (“diabetes” OR “diabetic” OR “hyperglycemia”)]. The search was limited to studies in humans. We only considered studies published as full-length articles in peer-reviewed journals in English or Chinese. As a supplementation, the references of related original and review articles were also manually screened for potentially related studies. The literature published from the inception of the databases to October 15, 2023, was screened.

### Inclusion and exclusion criteria

The inclusion criteria for the potential studies were: (1) observational studies published as full-length articles, (2) included women with GDM and healthy pregnant women, (3) measured the serum level of copeptin in women with and without GDM who were matched at least for gestational age (GA) of blood sampling, and (4) reported the difference of serum copeptin between women with and without GDM, or the difference could be calculated. The diagnostic criteria for GDM were in accordance with the criteria used among the included studies. No restriction was applied regarding the timing and methods for the measurement of serum copeptin.

Exclusion criteria were: (1) studies including pregnant women with other clinical conditions, such as pregnant-induced hypertension, preeclampsia, or pregestational diabetes, (2) studies including pregnant women with concurrent medications which may affect the blood glucose levels, such as corticosteroids, (3) studies that did not measure copeptin or did not compare serum copeptin between women with and without GDM, and (4) preclinical studies, reviews, or editorials. If studies with overlapping populations were retrieved, the one with the largest sample size was included in the meta-analysis.

### Study quality evaluation and data extraction

The processes of literature search, study identification, study quality evaluation, and data collection were independently conducted by two authors. If disagreement occurred, a consultation with the corresponding author was indicated to resolve the disagreement. We used the Newcastle–Ottawa Scale (NOS) [29] for the assessment of the quality of the included studies. This scale consisted of three aspects, including selection of cases and controls, comparability between groups, and exposure measurement. The total scores of NOS were 1–9, with 9 indicating the best quality. The following data was extracted from each study for subsequent analysis, including study information (author, year, country), participants’ characteristics (sample size, age, and body mass index (BMI) of the pregnant women), copeptin measuring (timing and methods), diagnosis of GDM (diagnostic criteria and number of women with GDM), and variables matched/adjusted when the association between serum copeptin and GDM was reported.

### Ethical statement

Ethical approval was not required for this study in accordance with local/national guidelines. Written informed consent to participate in the study was not required in accordance with local/national guidelines.

### Statistical analysis

A standardized mean difference (SMD) and corresponding 95% confidence interval (CI) was used to summarize the difference in serum copeptin levels between women with GDM and healthy pregnant women because the methods and units for measuring serum copeptin varied among the included studies [30]. The Cochrane *Q* test was performed to test the extent of heterogeneity between the studies, as well as the estimation of the statistic of I^2^ [30, 31]. Mild, moderate, or significant heterogeneity was considered if I^2^ < 25%, 25%–75%, or > 75%. We used a random-effects model to pool the results, which could incorporate the potential influences of between-study heterogeneity [28]. The sensitivity analyses by omitting one study at a time were performed to investigate the robustness of the findings. The predefined univariate meta-regression analyses were performed to evaluate the potential modification effects of study characteristics on the outcome, including sample size, mean maternal age, mean BMI, and NOS. The predefined subgroup analyses were also performed to evaluate the influences of study characteristics on the outcome, such as study country, mean maternal age, BMI, timing and methods for measuring serum copeptin, and criteria for the diagnosis of GDM. The medians of the continuous variables were used as the cutoffs for defining subgroups. The estimation of publication bias underlying the meta-analysis was first achieved by construction of the funnel plots and visual inspection of the plot symmetry [32]. An Egger’s regression test was also performed [32]. The statistical analysis was carried out using RevMan (Version 5.1; Cochrane Collaboration, Oxford, UK) and Stata software (version 12.0; Stata Corporation, College Station, TX, USA). A two-sided *P* < 0.05 suggests statistical significance.

## Results

### Study inclusion

**Figure 1.**
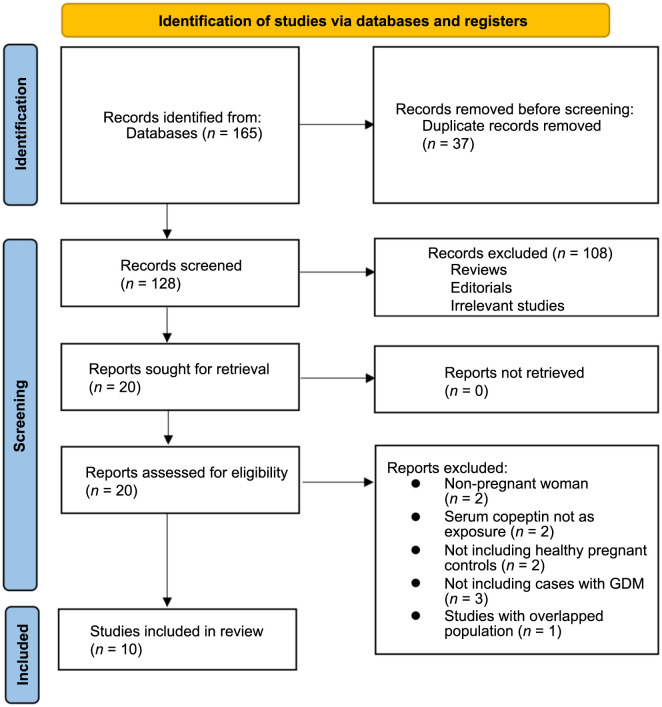
**The flowchart of database search and study inclusion.** GDM: Gestational diabetes mellitus.

The process of study inclusion is presented in Figure 1. In brief, 165 potentially relevant records were obtained after a comprehensive search of the 5 databases, and 37 of them were excluded due to duplication. Subsequently, a screening via titles and abstracts of the remained records further excluded 108 studies, mostly because they were not related to the aim of the meta-analysis. Accordingly, the full texts of the 20 left records were read by 2 independent authors, and 10 of them were further removed for the reasons listed in Figure 1. Finally, 10 observational studies were considered to be suitable for the subsequent quantitative analyses [16–25]. All of the included studies enrolled women without other comorbidities except GDM and without a medication history that may affect the blood glucose level.

### Overview of study characteristics

Table 1 represents the summarized characteristics of the included studies. Overall, 10 observational studies, including 9 case-control studies [16–21, 23–25] and 1 cross-sectional study [22] were included in the meta-analysis. These studies were published between 2013 and 2022, and performed in Turkey, Germany, Poland, and China. A total of 1837 pregnant women were included, including 625 women with GDM, and 1212 healthy pregnant women. The mean maternal age was 26.4–34.2 years, and the maternal BMI was 21.8–30.2 kg/m^2^. Blood sampling for measuring serum copeptin was at the early stage of pregnancy (GA: 8–2 weeks) in two studies [22, 23], at GDM screening (GA: 24–32 weeks) in six studies [18–21, 24, 25], and at or after delivery (GA: 37–39 weeks) in another two studies [16, 17]. Measuring serum copeptin was with the enzymatic immunoassay in three studies [16–18], with the illuminometric assay in two studies [19, 22], and with the enzyme-linked immunosorbent assay in the remaining five studies [20, 21, 23–25]. The diagnosis of GDM was in accordance with the American College of Obstetricians and Gynecologists criteria [33] in two studies [16, 17], and with the International Association of Diabetes in Pregnancy Study Group criteria [34] in the other eight studies [18–25]. Potential confounding factors, such as GA of blood sampling, were matched between women with and without GDM among all of the included studies, and some other variables, such as maternal age, BMI, blood pressure, and parity were also matched to a varying degree. The NOS of the included studies were seven to nine stars, suggesting overall good study quality (Table 2).

**Table 1:** Characteristics of the included studies

**Study**	**Country**	**Design**	**Women included (n)**	**Maternal age (years)**	**BMI (kg/m^2^)**	**Sampling time (weeks, range)**	**Mean GA at sampling (weeks)**	**Methods for measuring serum copeptin**	**Diagnosis of GDM**	**Number of women with GDM (n)**	**Variables adjusted**
Oncul 2013	Turkey	C-C	85	34.2	27.4	At delivery (GA: 37–39)	38	EIA	ACOG	45	Material age and GA
Aydin 2013	Turkey	C-C	30	28.7	30.2	After delivery (GA: 37–39)	38	EIA	ACOG	15	Maternal age, parity, maternal BMI, and GA
Ebert 2016	Germany	C-C	128	30	23.5	At GDM screening (GA: 24–28)	26	Illuminometric assay	IADPSG	74	Maternal age, BMI, and GA
Dabrowski 2016	Poland	C-C	58	31.3	28.2	At GDM screening (GA: 24–28)	26	EIA	IADPSG	40	Maternal age, BP, and GA
Wang 2016	China	C-C	92	28	23.1	At GDM screening (GA: 25–32)	28	ELISA	IADPSG	46	Maternal age, BMI, and GA
Ma 2017	China	CS	827	26.4	27.7	Before GDM screening (at first prenatal visit)	11	Illuminometric assay	IADPSG	101	GA
Chen 2017	China	C-C	90	28.3	23.3	At GDM screening (GA: 24–28)	26	ELISA	IADPSG	50	Maternal age, BMI, and GA
Ma 2019	China	C-C	256	32.3	21.8	Before GDM screening (GA: 8–12)	10	ELISA	IADPSG	128	Maternal age, BMI, and GA
Song 2021	China	C-C	172	27.6	23.8	At GDM screening (GA: 24–28)	26	ELISA	IADPSG	86	Maternal age, BP, BMI, and GA
Chen 2022	China	C-C	80	28.5	28.6	At GDM screening (GA: 24–28)	26	ELISA	IADPSG	40	Maternal age, BMI, and GA

BMI: Body mass index; GA: Gestational age; GDM: Gestational diabetes mellitus; C-C: Case-control study; CS: Cross-sectional study; EIA: Enzymatic immunoassay; ELISA: Enzyme-linked immunosorbent assay; ACOG: American College of Obstetricians and Gynecologists; IADPSG: International Association of Diabetes in Pregnancy Study Group; BP: Blood pressure.

**Table 2:** Study quality evaluation via the Newcastle-Ottawa Scale

	**Adequate definition of the cases**	**Representativeness of the cases**	**Selection of controls**	**Definition of controls**	**Controlled for GA**	**Controlled for other confounding factors**	**Ascertainment of the exposure**	**Same method of ascertainment of exposure for cases and controls**	**Non-response rate**	**Overall**
Oncul 2013	1	0	1	1	1	1	1	1	1	8
Aydin 2013	1	0	1	1	1	1	1	1	1	8
Ebert 2016	1	1	1	1	1	1	1	1	1	9
Dabrowski 2016	1	0	1	1	1	1	1	1	1	8
Wang 2016	1	0	1	1	1	1	1	1	1	8
Ma 2017	1	1	1	1	1	0	1	1	0	7
Chen 2017	1	0	1	1	1	1	1	1	1	8
Ma 2019	1	0	1	1	1	1	1	1	1	8
Song 2021	1	1	1	1	1	1	1	1	1	9
Chen 2022	1	1	1	1	1	1	1	1	1	9

GA: Gestational age.

### Results of the meta-analysis

**Figure 2.**
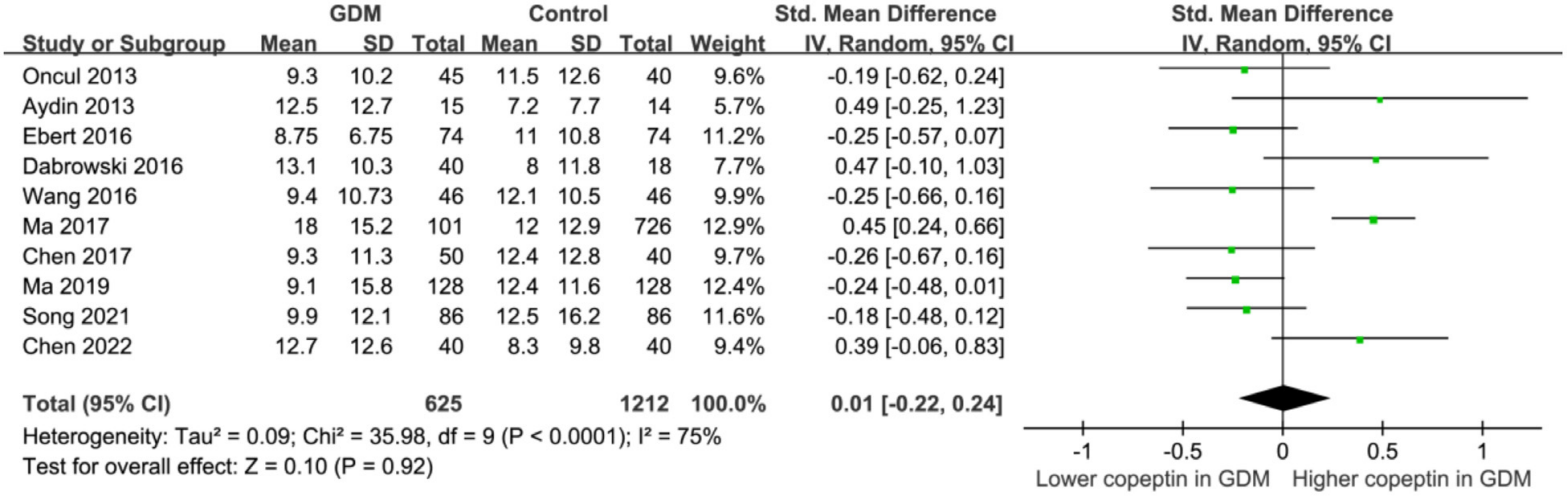
**Forest plots for the meta-analysis comparing serum copeptin between women with and without GDM.** GDM: Gestational diabetes mellitus; SD: Standard deviation; CI: Confidence interval; Chi^2^: Chi square.

**Figure 3.**
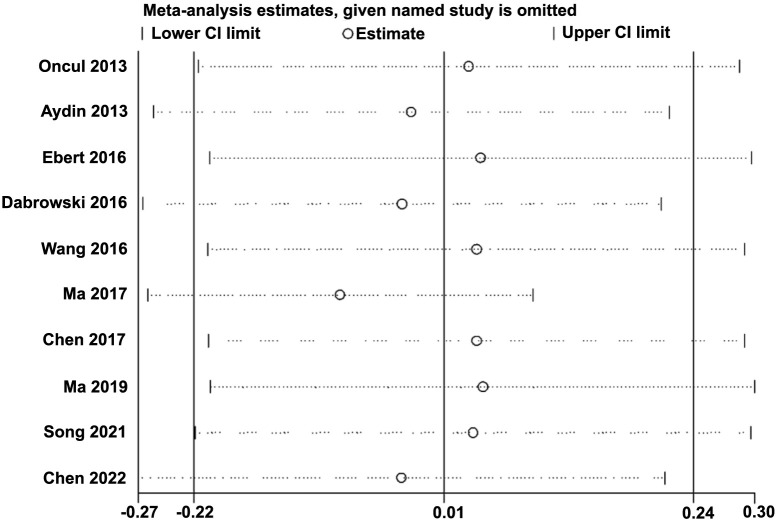
**Plots showing the results of sensitivity analysis by excluding one study at a time.** CI: Confidence interval.

The heterogeneity was significant among the included studies (*I*^2^ ═ 75%). Pooled results with a random-effects model showed that the serum copeptin was not significantly different between women with and without GDM (SMD 0.01, 95% CI −0.22 to 0.24, *P* ═ 0.92, Figure 2). Sensitivity analysis by excluding one dataset at a time did not significantly affect the results (*P* all > 0.05, Figure 3).

**Figure 4.**
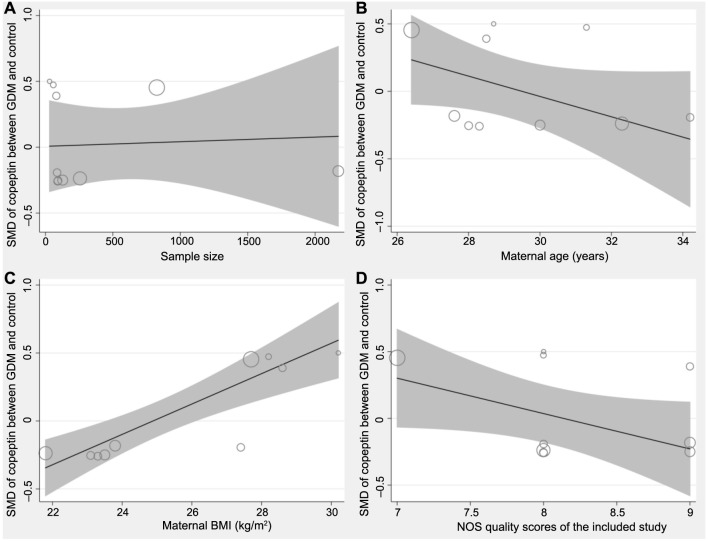
**Plots showing the results of univariate meta-regression analysis.** Meta-regression for the influence of (A) sample size; (B) mean age; (C) maternal BMI and (D) NOS. BMI: Body mass index; NOS: Newcastle-Ottawa Scale; SMD: Standardized mean difference; GDM: Gestational diabetes mellitus.

The univariate meta-analysis did not support that sample size (coefficient ═ −0.00003, *P* ═ 0.86, Figure 4A) or mean maternal age (coefficient ═ −0.047, *P* ═ 0.33, Figure 4B) which might significantly affect the results of the meta-analysis. Interestingly, the maternal BMI was shown to positively modify the results (coefficient ═ 0.11, *P* ═ 0.002, Figure 4C), which largely explained the heterogeneity (residual *I*^2^ ═ 1.06%). A further meta-regression analysis also failed to show that NOS may significantly affect the results (coefficient ═ −0.18, *P* ═ 0.30, Figure 4D).

**Figure 5.**
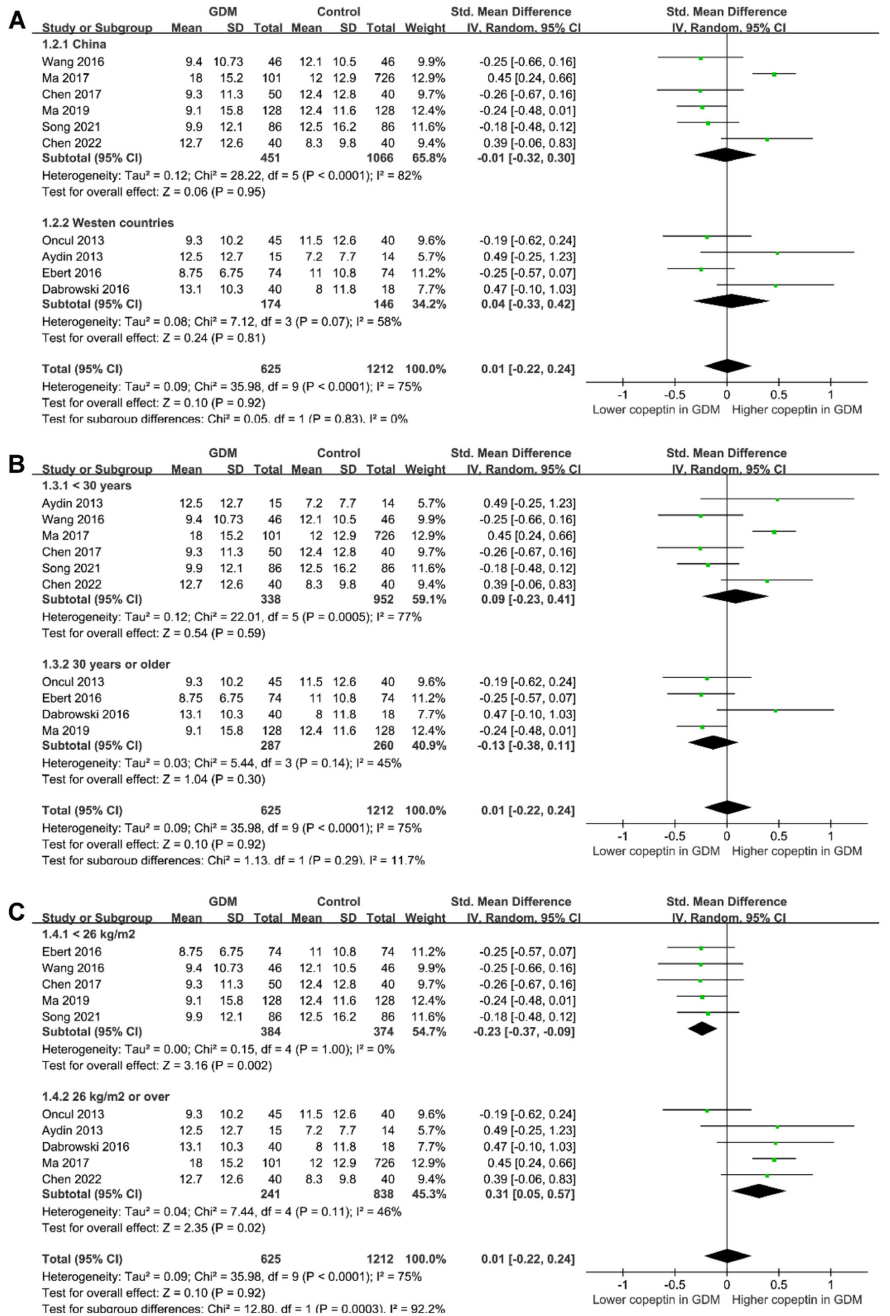
**Forest plots for the subgroup analyses comparing serum copeptin between women with and without GDM.** (A) Subgroup analysis according to study country; (B) Subgroup analysis according to mean age; and (C) Subgroup analysis according to maternal BMI. GDM: Gestational diabetes mellitus; BMI: Body mass index; SD: Standard deviation; CI: Confidence interval; Chi^2^: Chi square.

**Figure 6.**
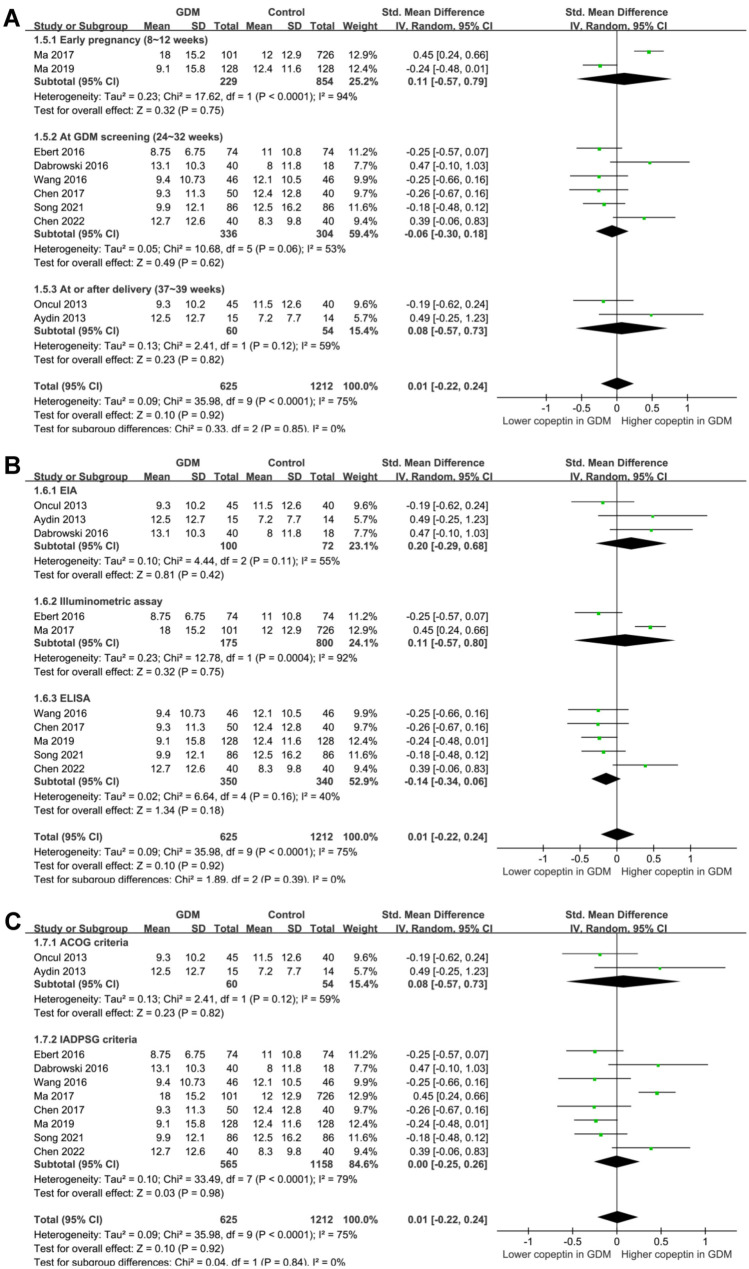
**Forest plots for the subgroup analyses comparing serum copeptin between women with and without GDM.** (A) Subgroup analysis according to the timing of blood sampling; (B) Subgroup analysis according to the methods for measuring copeptin and (C) Subgroup analysis according to the diagnostic criteria for GDM. GDM: Gestational diabetes mellitus; SD: Standard deviation; CI: Confidence interval; Chi^2^: Chi square.

The results of subgroup analyses are shown in Figures 5 and 6. Although the results did not support that study country (*P* for subgroup difference ═ 0.83, Figure 5A), maternal age (*P* for subgroup difference ═ 0.29, Figure 5B), the timing of blood sampling for copeptin measuring (*P* for subgroup difference ═ 0.85, Figure 6A), methods for copeptin measuring (*P* for subgroup difference ═ 0.39, Figure 6B), or diagnostic criteria of GDM (*P* for subgroup difference ═ 0.84, Figure 6C) might significantly affect the association between serum copeptin and GDM, maternal BMI might significantly affect the results (*P* for subgroup difference ═ 0.0003, Figure 5C). Specifically, GDM was associated with a higher serum copeptin in women with mean BMI ≥ 26 kg/m^2^ (SMD 0.31, 95% CI 0.05–0.57, *P* ═ 0.02, *I*^2^ ═ 46%), but not in women with BMI < 26 kg/m^2^ (SMD −0.23, 95% CI −0.37 to −0.09, *P* ═ 0.002, *I*^2^ ═ 0%).

### Publication bias evaluation

**Figure 7.**
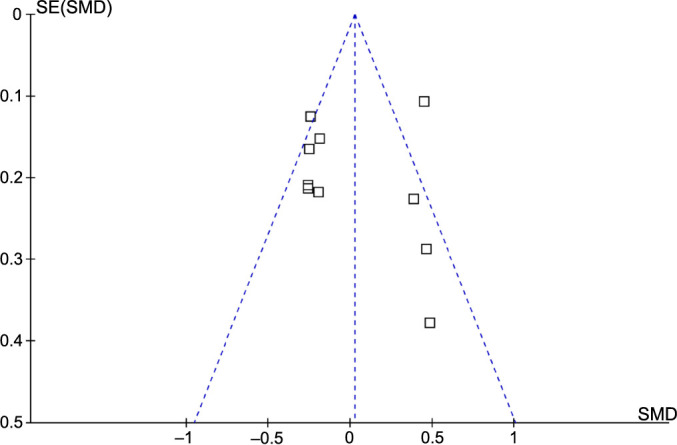
**Funnel plots for evaluating the possible publication bias of the meta-analysis comparing serum copeptin between women with and without gestational diabetes mellitus.** SE: Standard error; SMD: Standardized mean difference.

Figure 7 shows the funnel plots for the meta-analysis of the potential difference of serum copeptin between women with and without GDM. The plots were symmetrical on visual inspection, indicating a low risk of publication bias. Additionally, the results of Egger’s regression test also did not suggest a significant publication bias (*P* ═ 0.48).

## Discussion

In this meta-analysis, by combining the results of 10 available observational studies, the results showed that there was no significant difference in copeptin serum level between women with and without GDM. However, considerable heterogeneity was observed among the included studies. Further meta-regression and subgroup analysis suggested that the association between serum copeptin and GDM might be significantly modified by the BMI of the included women, which largely explained the between-study heterogeneity. Specifically, a high serum copeptin in GDM could be observed in pregnant women with BMI ≥ 26 kg/m^2^, but not in women with smaller BMI < 26 kg/m^2^. Taken together, these results suggest that BMI might significantly modify the association between serum copeptin and GDM, and a high serum copeptin might be correlated with GDM in women with BMI ≥ 26 kg/m^2^.

To the best of our knowledge, this study might be the first meta-analysis that summarized the changes of serum copeptin in women with GDM. A few mythological strengths should be noticed. First, we performed an extensive literature search in five commonly used English and Chinese databases, which retrieved ten up-to-date observational studies according to the aim of the meta-analysis. Second, in all of the included studies, the GA of blood sampling for measuring copeptin was matched between cases and controls. This is particularly important because it has been confirmed in previous studies that serum level of copeptin in pregnant women was GA dependent. Third, we performed sensitivity analyses by excluding one study at a time, and similar results confirmed the robustness of the finding, which was not primarily driven by single included studies. Finally, in view of the significant heterogeneity observed among the included studies, we performed multiple metaregression and subgroup analyses to explore the source of the heterogeneity. The results consistently suggested that the BMI of the women might positively affect the difference of serum copeptin between women with and without GDM, and a high serum copeptin might be correlated with GDM in women with BMI ≥ 26 kg/m^2^. On the other hand, the results of the meta-analysis were not significantly affected by other study characteristics, such as sample size, mean maternal age, study country, timing and methods for measuring copeptin, diagnostic criteria for GDM, or study quality scores. Taken together, the results of the meta-analysis suggested that a high serum copeptin might be associated with GDM in pregnant women with BMI ≥ 26 kg/m^2^.

A few hypotheses have been raised regarding the potential association between a high copeptin and GDM. As a precursor of AVP with equimolar concentration, an elevated copeptin serum level indicates a heightened systemic burden of AVP. AVP enhances the effects of corticotrophin-releasing hormone at the pituitary level, leading to an augmented secretion of adrenocorticotropic hormone and subsequently an increased release of cortisol that evades the inhibitory feedback mechanism [35]. Additionally, AVP governs the secretion of insulin and glucagon via the V1b receptor, while also stimulating hepatic glycogenolysis and gluconeogenesis through the V1a receptor [36]. Furthermore, AVP has been implicated in the pathogenesis of diabetic complications, including cardiovascular diseases, kidney complications, and ocular complications, in addition to its detrimental effects on glucose metabolism [37]. The assay of AVP poses significant challenges due to its minimal presence in the bloodstream, as well as its small size and limited stability [38]. Consequently, copeptin, the stable C-terminal fragment of the prepro-vasopressin peptide, has emerged as a more feasible alternative for measuring AVP activity [39].

Results of the meta-regression and subgroup analysis suggested that the association between copeptin and GDM might be affected by BMI of the women and that a high copeptin was positively associated with GDM in women with BMI ≥ 26 kg/m^2^. The mechanisms remain unknown. Interestingly, a previous preclinical study suggested that high-AVP promoted hyperinsulinemia and glucose intolerance in obese rates, but not in lean rats, suggesting that the pro-diabetic role of AVP might be more remarkable in obese rats [40]. These findings are consistent with the results of meta-regression and subgroup analyses in our meta-analysis. Although further studies are needed to determine potential mechanisms, the findings of the meta-analysis highlight the importance of considering the influence of BMI of women when the association between copeptin and GDM was investigated.

This study has limitations. First, the number of available studies for the meta-analysis is limited, and the results should be validated in large-scale studies. In addition, only case-control and cross-sectional studies were included, prospective cohort studies are needed to determine the longitudinal relationship between serum copeptin and GDM, particularly in women with BMI ≥ 26 kg/m^2^. Moreover, we could not exclude the residual uncontrolled factors that might affect the association between serum copeptin and GDM. Additionally, the methods for copeptin assays varied among the included studies, which may affect the results of the meta-analysis. Finally, as a meta-analysis of observational studies, a causative relationship between a high serum copeptin and GDM could not be established on the basis of the results.

## Conclusion

In conclusion, although the overall meta-analysis did not show a significant difference in serum copeptin between women with and without GDM, further meta-regression and subgroup analysis suggested that the association between serum copeptin and the risk of GDM might be modified by the BMI of the pregnant women. A high serum copeptin might be correlated with GDM in pregnant women with BMI ≥ 26 kg/m^2^, but not in those with BMI < 26 kg/m^2^. Further research is warranted to elucidate potential mechanisms. Nonetheless, the results of the meta-analysis highlight the importance of the influence of BMI on the association when the relationship between copeptin and GDM is investigated.

## Data Availability

All the data generated during the study are within the manuscript.
